# Oseltamivir phosphate released from injectable Pickering emulsions over an extended term disables human pancreatic cancer cell survival

**DOI:** 10.18632/oncotarget.24339

**Published:** 2018-01-29

**Authors:** Kurt Wood, Myron R. Szewczuk, Dérick Rousseau, Ronald J. Neufeld

**Affiliations:** ^1^ Department of Chemical Engineering, Queen's University, Kingston, Ontario K7L3N6, Canada; ^2^ Department of Biomedical and Molecular Sciences, Queen's University, Kingston, Ontario K7L3N6, Canada; ^3^ Department of Chemistry and Biology, Ryers on University, Toronto, Ontario M5B 2K3, Canada

**Keywords:** Pickering emulsion, sustained release, oseltamivir phosphate, competitive adsorption

## Abstract

Pickering emulsions are colloidal dispersions stabilized by particles that either migrate to, or are formed at, the oil-water interface during emulsification. Here, we fabricated and characterized Pickering water-in-oil emulsions where molten glycerol monostearate crystallized at the surface of micron-sized water droplets and formed protective solid shells. We tested this emulsion as a reservoir delivery platform for the sustained release of low molecular weight hydrophilic molecules including sodium chloride (NaCl) and sodium citrate as model compounds, and the therapeutic oseltamivir phosphate (OP), the delivery of which was the ultimate goal of this research. The objective was to achieve long-term (30-day) release of challenging to encapsulate actives and ultimately demonstrate the sustained release of OP for 20–30 days from an injectable formulation. OP was used because of its anticancer properties targeting mammalian neuraminidase 1 (Neu1) involved in multistage tumorigenesis. All actives including OP encapsulated in Pickering emulsions displayed a near linear release profile over 30 days. It was demonstrated that the release could be modulated by the addition of a second, competing surfactant sorbitan monooleate, Span 80, to the emulsion at levels above its critical micelle concentration. OP released from the emulsions significantly reduced cell viability in the human PANC-1 pancreatic cancer cell line for up to 30 days. The findings from this study indicate a simple, potentially injectable formulation and method that is easily upscaled resulting in a stable product with the potential to fully retain small hydrophilic molecules/drugs for sustained, near linear release over days, weeks, and potentially months.

## INTRODUCTION

Emulsions consist of two immiscible fluids where one is dispersed as discrete droplets within the other. Particle-based emulsion stabilization is increasingly being sought in place of more commonly used small-molecule or polymeric surfactants, owing to the ability of particles to confer remarkable resistance against droplet coalescence in both oil-in-water (O/W) and water-in-oil (W/O) emulsions. Given their entrapment within a deep energy well, interfacially-bound particles provide steric hindrance that may reduce emulsion sensitivity to temperature variations, pH and osmotic gradients. Such emulsions are denoted as Pickering emulsions, based on one of their first reports [1].

There is growing interest in the use of particle-stabilized emulsions for applications requiring stability in complex applications such as personal care products, drug delivery, and processed foods because of their elegant simplicity and controlled release properties. For example, Simovic *et al.* [2] found that interfacially-bound nanoparticle layers significantly influenced the release kinetics of lipophilic drugs from Pickering-based O/W emulsions. Liu *et al.* [3] have shown that Pickering emulsions stabilized by soy protein-based particles performed as sustained release delivery systems for β-carotene. Pickering emulsions have also been highly useful in topical drug delivery systems [4–6].

One of the challenges faced in the area of controlled delivery systems involves the full encapsulation, retention, and controlled long-term sustained release of small molecular weight hydrophilic compounds. We have previously demonstrated that implantable double-layered poly(D, L-lactic-co-glycolic acid) (PLGA) cylinders engineered to sequentially release the hydrophilic chemotherapeutic drug, gemcitabine (GEM), in combination with oseltamivir phosphate (OP) over an extended time had minimal loss of drugs during the formulation [7]. This protocol enabled extensive control of drug loading and establishing uniform drug distribution throughout the polymer matrix. The controlled release of OP from the biodegradable PLGA cylinder (PLGA-OP) implanted at the tumor site was investigated for its role in limiting tumor neovascularization, growth, and metastasis [8]. The PLGA-OP cylinders over 30 days *in vitro* had approximately 20%–25% release profiles within 48 hours followed by a continuous metronomic low dose release of 30–50% OP for an additional 16 days with complete OP released by day 30. Surgically implanted PLGA-OP containing 20 mg OP and empty PLGA cylinders near the tumor site of heterotopic xenografts of human pancreatic PANC1 cancer cells in RAGxCγ double mutant mice impeded tumor neovascularization, growth rate, and metastatic spread to the liver and lungs compared with the untreated cohort [8]. Here, OP is used in the formulation because of its anticancer drug properties targeting mammalian neuraminidase 1 (Neu1) involved in multistage tumorigenesis [9] for pancreatic [10], triple negative breast [11] and ovarian [12] cancers. To this end, we engineered and developed OP-conjugated polymeric micelles prepared by RAFT living radical polymerization to target Neu1, which specifically bound Neu1 and activated receptor endocytosis and OP-micelle internalization [13]. The OP-micelle and monoglyceride-based Pickering-stabilized emulsion (MSPE) delivery systems as described in the present study are of interest because of their injectable formulation properties as opposed to implantable ones. The OP conjugated-micelle formulation is chemically impressive because of its unique targeting and internalization properties, but the chemistries are complicated and time-consuming. On the other hand, the MSPE approach is simple and straightforward, using equipment and reagents that are routinely found in a pharmacy with the formulation time under 1h, and involving no chemistry.

The invasive nature of drug delivery implants and poor outcomes with alternative systemic chemotherapy approaches has led to the development of an intraperitoneal injectable formulation with similar extended term release characteristics to the PLGA implant, avoiding the need for implantation or use of intraperitoneal catheters for drug administration. Here, we propose that an injectable Pickering emulsion bolus would function as a “soft implant” delivered through injection, and guided by imaging methods such as endoscopic ultrasound directly to the pancreatic tumor site. Both intra- and extra-tumor options are possible as chemotherapeutic approaches followed by sustained delivery of the chemotherapeutic over an extended period, which would represent a single dosage with the minimally invasive therapeutic approach. Because of the viscous nature of the Pickering emulsion, the bolus is expected to remain at the site of injection (implantation) and not diffuse or disperse throughout the tissue, thus better replicating the outcomes achieved with the solid implant.

The use of Pickering emulsions for drug delivery is a new and growing field, but applications in the food area are more extensively developed. The primary goal in the present study was to develop a delivery vehicle for OP as an injectable formulation, which was significantly facilitated through the developmental work and comparisons to model compounds such as sodium chloride and sodium citrate, both of which are hydrophilic and of low molecular weight like OP. Here, we describe the retention and sustained release behavior of the two model compounds and OP from Pickering emulsions stabilized with glycerol monostearate (GMS) crystalline shells. The additional presence of sorbitan monooleate as a secondary surfactant permitted controlled destabilization of the GMS crystalline layer, enabling the potential to modulate active release from the Pickering-stabilized emulsion. In the present study, OP released from the Pickering emulsion remained therapeutically active in reducing viability of human pancreatic cancer cells over an extended release period of 30 days.

## RESULTS AND DISCUSSION

The primary objective of this research was the design of an injectable drug delivery vehicle for targeted and extended-term delivery of oseltamivir phosphate (OP) against pancreatic cancer. In the present study, a modified W/O monoglyceride-stabilized Pickering emulsion (MSPE) construct is based on the encapsulation, and long-term sustained release of small molecular weight hydrophilic molecules such as NaCl in food applications [14]. The MSPE formulation was tested and optimized using low molecular weight hydrophilic solutes, as a precursor to the application involving the OP therapeutic effect on cancer cells. Solutes used in the development of the MSPE system were NaCl and Na-citrate, based on their low molecular weight as salt, and an organic acid salt, respectively. Both of these compounds are of interest for a variety of industrial applications in food ingredients, pharmaceuticals, and personal care products. The extension to incorporate OP in the formulation of MSPE was examined in the present study for its long-term stability and release to disable human cancer cells survival as previously described [7, 8, 11, 13, 15, 16].

**Figure 1:**
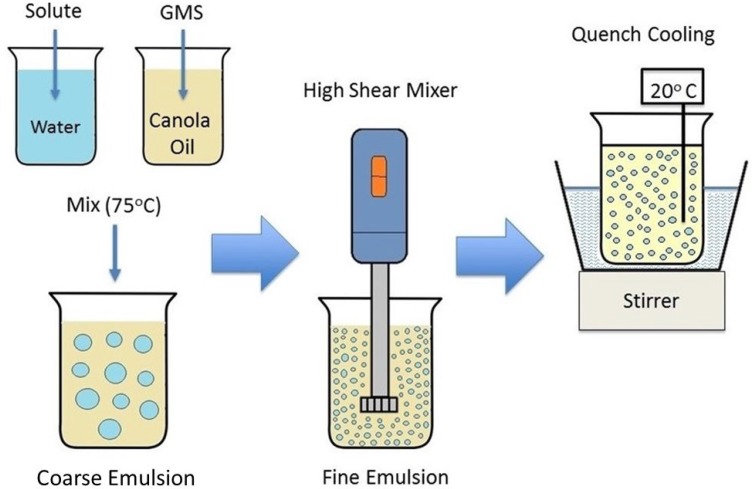
Formulation of W/O monoglyceride stabilized Pickering emulsions

**Figure 2:**
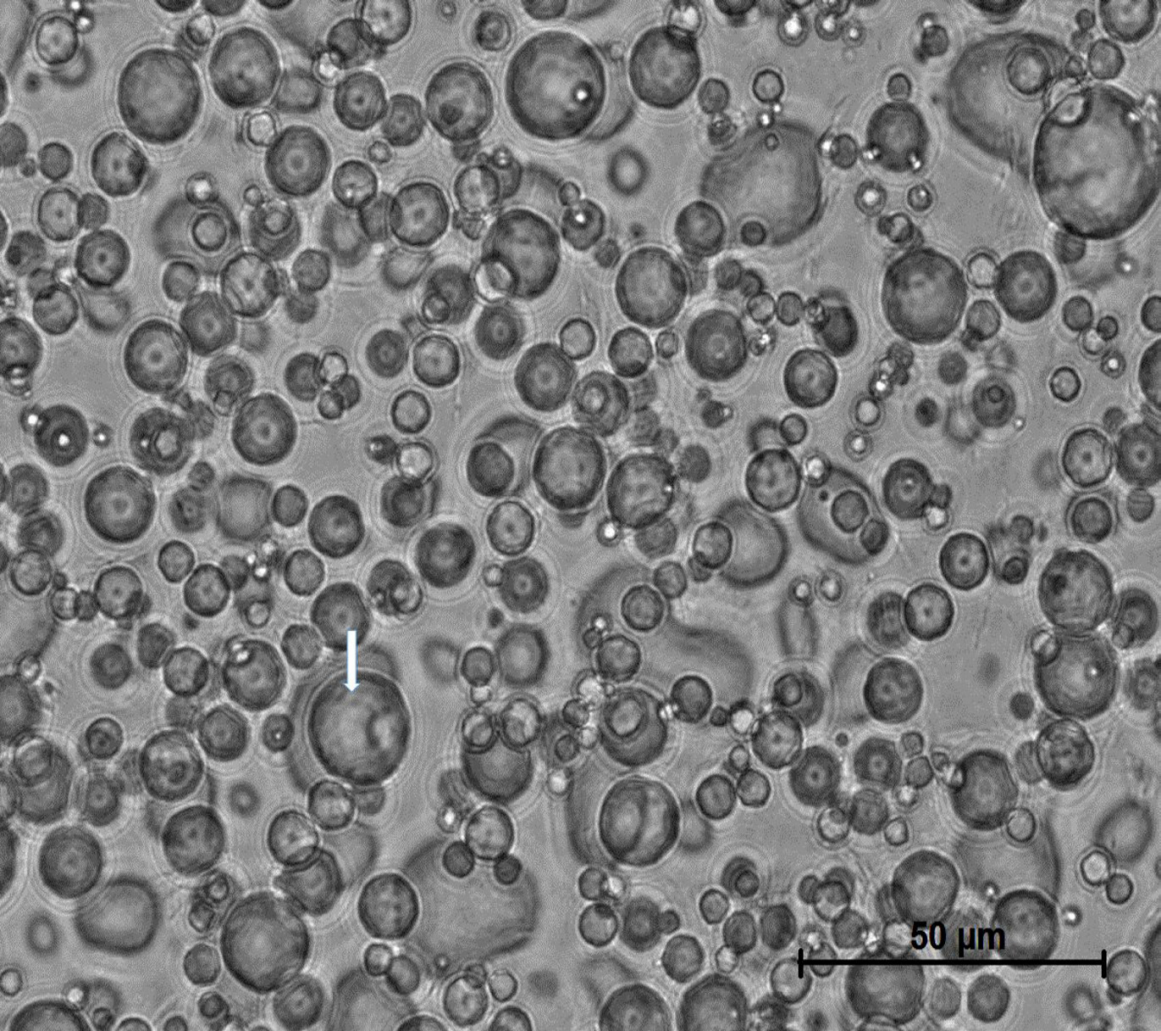
Micrograph of MSPE Mean droplet size was 10 μm. Dimpled surface on all of the emulsion droplets such as the one highlighted with the arrow is evidence for GMS crystallization on the droplet surface.

The Pickering emulsion approach as illustrated in Figure 1 and described in detail in the Methods section, was adapted from Nadin *et al.* [14] and formulated as a W/O system stabilized by GMS. In the molten state, GMS adsorbs to the oil-water interface, and upon cooling, interfacial solidification occurs, resulting in crystalline shells encasing the aqueous emulsified droplets in a continuous oil phase. Briefly, during homogenization at high temperature where the GMS is molten, adsorption of emulsifier molecules at the oil-water interface will occur with their polar moieties oriented towards the dispersed aqueous phase and hydrophobic groups towards the oil phase. The resulting hairy brush layer will comprise interfacially-anchored gauche stearic chains generally organized to the interface. With a drop in temperature, the GMS stearic acid chains will undergo a gauche-trans conversion resulting in heterogeneous nucleation at the droplet surface leading to the formation of interfacially-bound crystal nuclei tethered to the drop surface. The continuous stirring of the emulsion during cooling was essential to provide rapid, uniform GMS solidification, lessen the extent of aqueous droplet flocculation, and prevent settling of the dispersed phase. The oil-water weight ratio was 80/20. The practical range of dispersed phase fraction was determined to be ≤ 30 wt%, beyond which emulsion viscosity became high enough to hinder mixing. At 50%, formulations became unstable, displaying a tendency to invert. At 70% aqueous phase, formulations inverted from W/O to O/W emulsions with significantly lower viscosity. The interfacial growth of the GMS crystals will proceed as the nascent crystals thicken, spread and finally cover the whole drop with a shell, until which they are constrained by either droplet curvature or full droplet coverage. The GMS crystals will start off as discrete particles where initial nuclei are formed. These particles will catalyze the solidification process of neighboring GMS molecules. Eventually, growing crystals will abut each other, at which point growth will stop. The grain boundaries between adjacent crystals will form, resulting in imperfections at the MSPE droplet surface as depicted by the arrow in the photomicrograph in Figure 2. The emulsion droplet size was controlled through the mixing rate process during emulsification and was stable for tested emulsions for at least one year with little change in droplet size.

**Figure 3:**
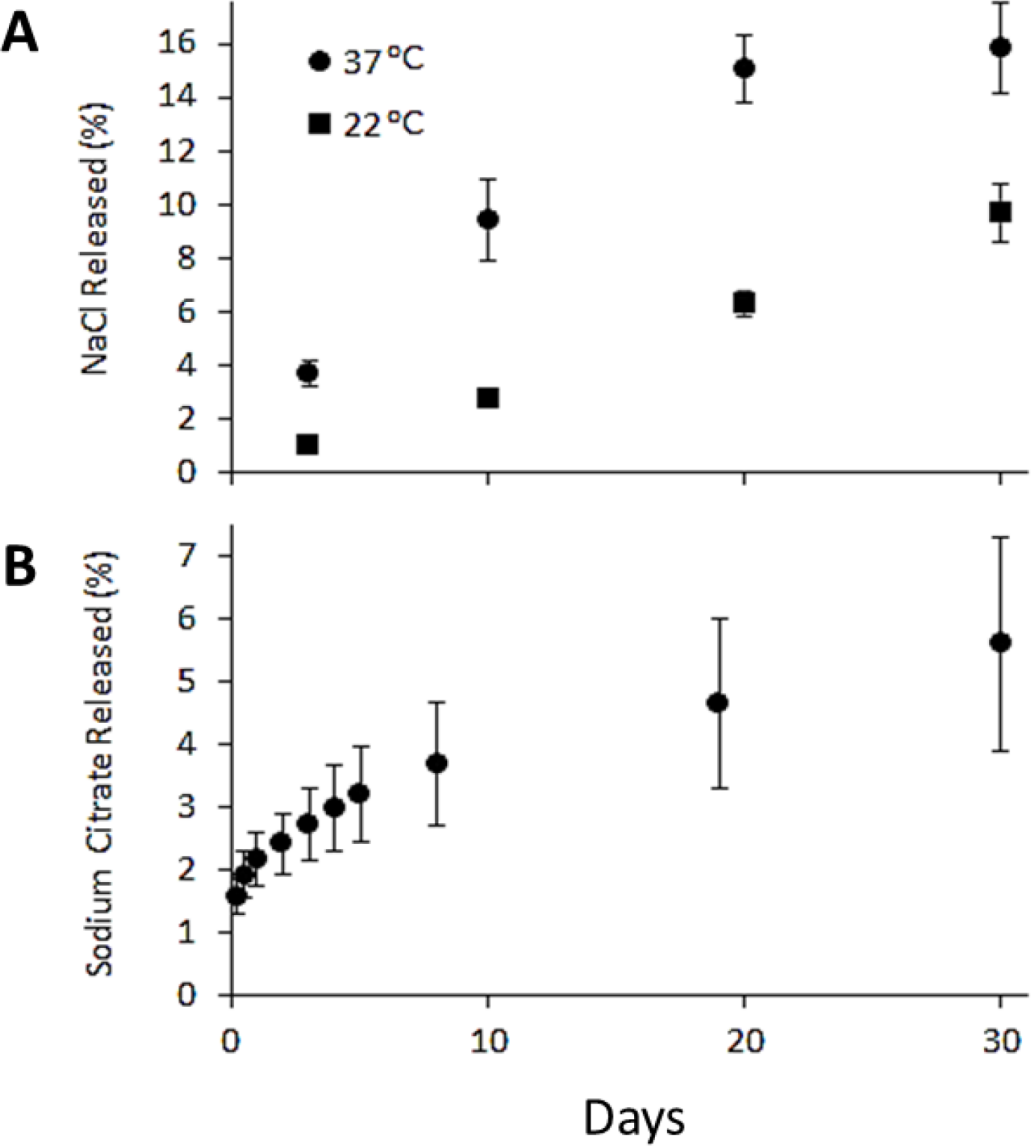
Release profiles (**A**) Sodium chloride at 22 and 37° C over 30 day period to simulate ambient and physiological conditions, respectively. Results are given for solute release as a percent of initial loading of solute in the emulsion. (**B**) Sodium citrate over 30 day period as a percent of initial loading of solute in the emulsion.

Previously, the release of NaCl from an MSPE was assessed over a few hours [14]; however, in the present study, NaCl encapsulated in GMS-MSPE was assessed over 30 days (Figure 3A) which is consistent with the objective to release therapeutic agents such as OP.

A slow, sustained release of NaCl was observed over 20 to 30 days, with between 10 and 16% of the NaCl released depending on temperature over that period. At 22°C, the release profile appeared nearly linear, particularly over the 10 to 30 day period, and at 37°C, the release profile was linear up to day 20, followed by an exponential decay and plateau between days 20 and 30. The appearance of linear release profiles over most of the release period was consistent with that observed by Nadin *et al.* [14] over a 2 h release period of NaCl from a GMS-stabilized Pickering emulsion.

Following 30 day release trials, emulsions were broken down to measure the amount of retained salt. This approach was achieved by dispersing the emulsion in water and heating it to melt the GMS stabilizer, which liberated the remaining entrapped solute. The cumulative mass of NaCl released over 30 days, when combined with the mass of salt released upon the final breakdown of the emulsion, yielded a total mass of salt which was typically within 5% of the initial amount of salt used to formulate the emulsion. Thus, it is apparent that the MSPE provided essentially full initial encapsulation of the low molecular weight hydrophilic solute, as all of the solute was entirely accounted for at termination of the experiment.

For comparison, release profiles were obtained for similar MSPEs by encapsulating Na citrate, which was selected as a good proxy for drug release behavior since OP is the salt of an organic base that is close in molecular weight to citrate. As was the case for NaCl emulsions, Na citrate was added to the MSPE at 4% in the aqueous phase. The data shown in Figure 3B indicated a slow, sustained release of Na citrate from the MSPEs over a 30-day period at 37°C, approaching 6% of the total solute loading. The release profile showed a decaying release rate, plateauing to near zero release by day 20. The same behavior was observed in formulations encapsulating NaCl. However, the fraction of total solute released over 30 days was more substantial. It is apparent from the release profiles of NaCl and Na citrate that sustained release of a small hydrophilic molecule can be achieved over time periods of 20 to 30 days, while avoiding an initial burst release. The solute release from a Pickering emulsion was not dependent on solute diffusion since the solutes tested in this case were not oil-soluble. Emulsion stability was likely to be the primary driver of solute release, suggesting that the triggers destabilizing Pickering emulsions may enhance the release rate, and if controllable, would provide the ability to modulate release. Several options are available for emulsion destabilizers, but because the actives under consideration in this study were intended for food and pharmaceutical application, only food-grade or physiologically compatible destabilizers were considered.

**Figure 4:**
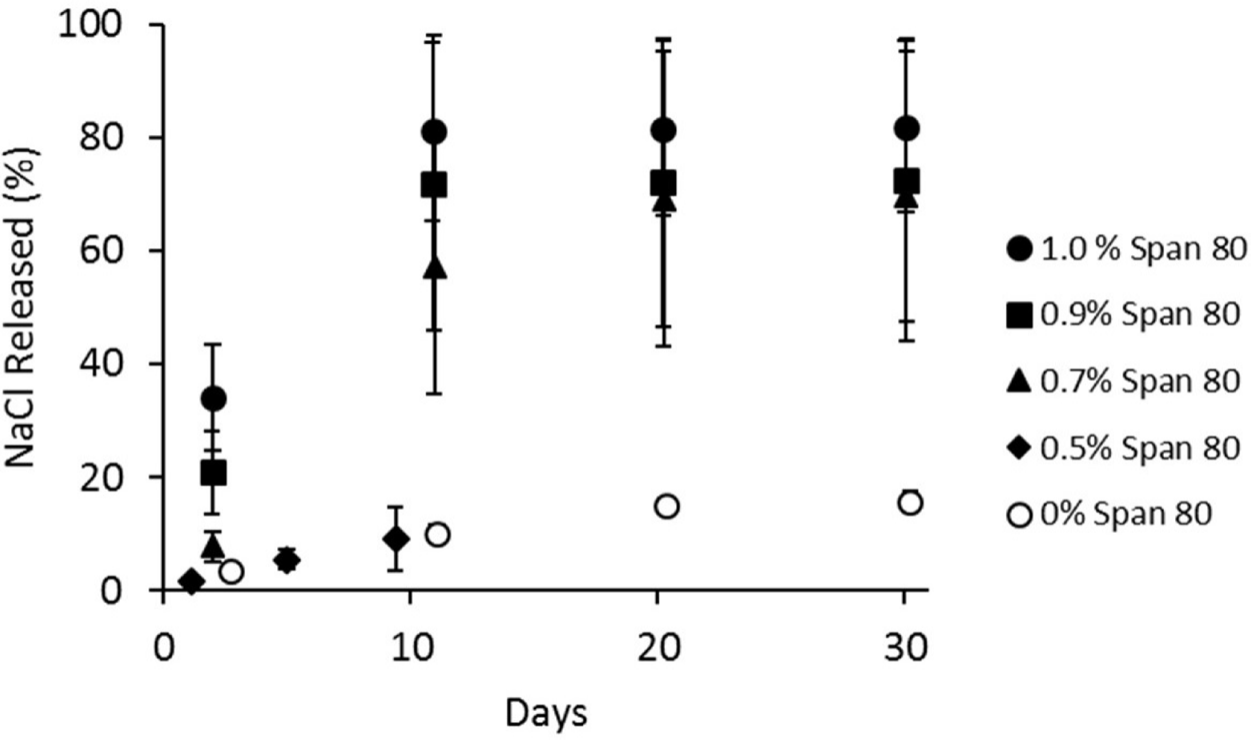
Release profiles of NaCl at 37° C for MSPE formulations containing Span 80 added post-emulsification Span 80 concentration is expressed as wt% of the oil phase. Results are given as percent of total NaCl released.

Formulations containing the nonionic surfactant sorbitan monooleate, Span 80 were prepared to examine the impact of oil phase amphiphiles on GMS-based MSPE emulsion stability and release behavior. MSPEs, as described, were prepared with NaCl as active, but with Span 80 in oil added following emulsification and solidification of the GMS. Blank formulations containing 1% Span 80 were prepared to act as a control. The release profiles for formulations containing Span 80 as emulsion destabilizer are shown in Figure 4.

At Span 80 concentrations below 0.7%, there was little effect on release behavior, which was comparable to formulations that did not contain any Span 80. Similar results were obtained for formulations with 0.1, 0.3 and 0.6% Span 80 (data not shown). Formulations containing Span 80 at a concentration ≥0.7% resulted in significantly elevated solute release rates for the initial ten days of release. At 0.7, 0.9, and 1.0% Span 80, 70, 73 and 82% of the total salt loading, respectively, were released over 30 days. This approach implies that a concentration of 0.7% Span 80 was the threshold resulting in destabilization of the MSPE and enabling enhanced release of the solute.

Addition of Span 80 pre- or post-emulsification resulted in statistically similar release profiles. The effect of temperature on release was also demonstrated using formulations containing 1% Span 80. The release profiles were sharply different, with 28 and 82% of the NaCl released over 30 days at 22 and 37°C, respectively. The more significant NaCl release observed at higher temperature was presumably related to the higher thermal energy of the interfacially-bound GMS crystals and liquid-state Span 80, which accelerated the diffusion, displacement, and desorption of GMS from the oil-water interface. It was not related to the melting point of GMS (54–64°C), which is well above 37°C.

**Figure 5:**
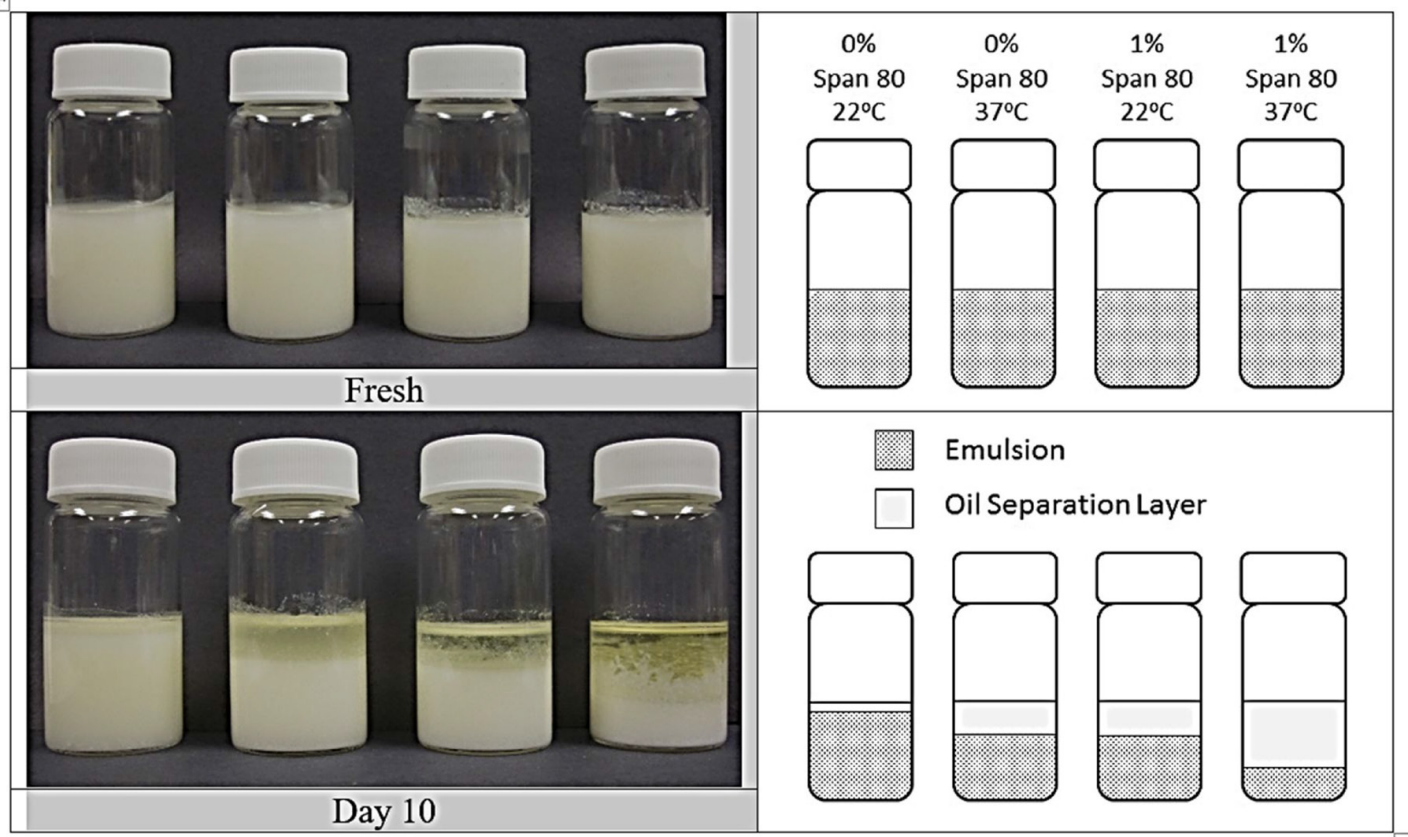
Stability of MPSE without solute added, but containing 0 or 1% Span 80 in the oil phase, as compared for freshly prepared emulsions (top) and emulsions after ten days (bottom) The content and storage conditions of each vial are shown in the top right panel, and a to-scale depiction of the resulting settling behavior is given in the top and bottom right panel.

Visual examination of MPSE showed emulsion instability as a mechanism for solute release. Visual phase separation was not evident as shown in Figure 5 for emulsions without Span 80 present (upper panel), but sharply evident after ten days for emulsions in the presence of Span 80 (lower panel). As well, phase separation was more strongly evident at 37°C compared to 22°C. Phase separation appeared consistent with the increased rate of solute release as presented in Figure 4 for NaCl release in the presence of Span 80 and strongly suggested that emulsion droplet flocculation and coalescence were driving the physical breakdown of the emulsion.

Several mechanisms for the destabilization of Pickering emulsions upon surfactant addition have been previously proposed. In surfactant-stabilized emulsions, the introduction of a new surfactant potentially results in a competition for the interface, shifting the interface-bulk equilibrium of both surfactants [17], with the final interfacial stabilizer composition determined by the relative affinity of each agent for the surface versus the bulk phase. Under the present experimental conditions, GMS was a solid-state material, firmly bonded to the droplet surface, with the energy required for removal orders of magnitude larger than that required for liquid surfactants. There have been reported instances of Pickering particle stabilizers being displaced from droplet interfaces due to the addition of surfactants. Notably, Vilchez *et al.* [18] showed that surface-modified magnetite nanoparticles were removed from droplet surfaces in a W/O emulsion due to the addition of a lipophilic surfactant. It was demonstrated that the surfactant adsorbed to the surface of the nanoparticles, increasing hydrophobicity until the particles were too hydrophobic to adhere to the droplet surface, at which point they became dispersed in the continuous oil phase. It is possible that this could also occur in the GMS-stabilized Pickering emulsions with the Span 80 adsorbing onto the surface of the GMS crystals, thereby altering their wetting behavior and ability to adsorb onto the oil-water interface, thus rendering them ineffective as emulsion stabilizers.

**Figure 6:**
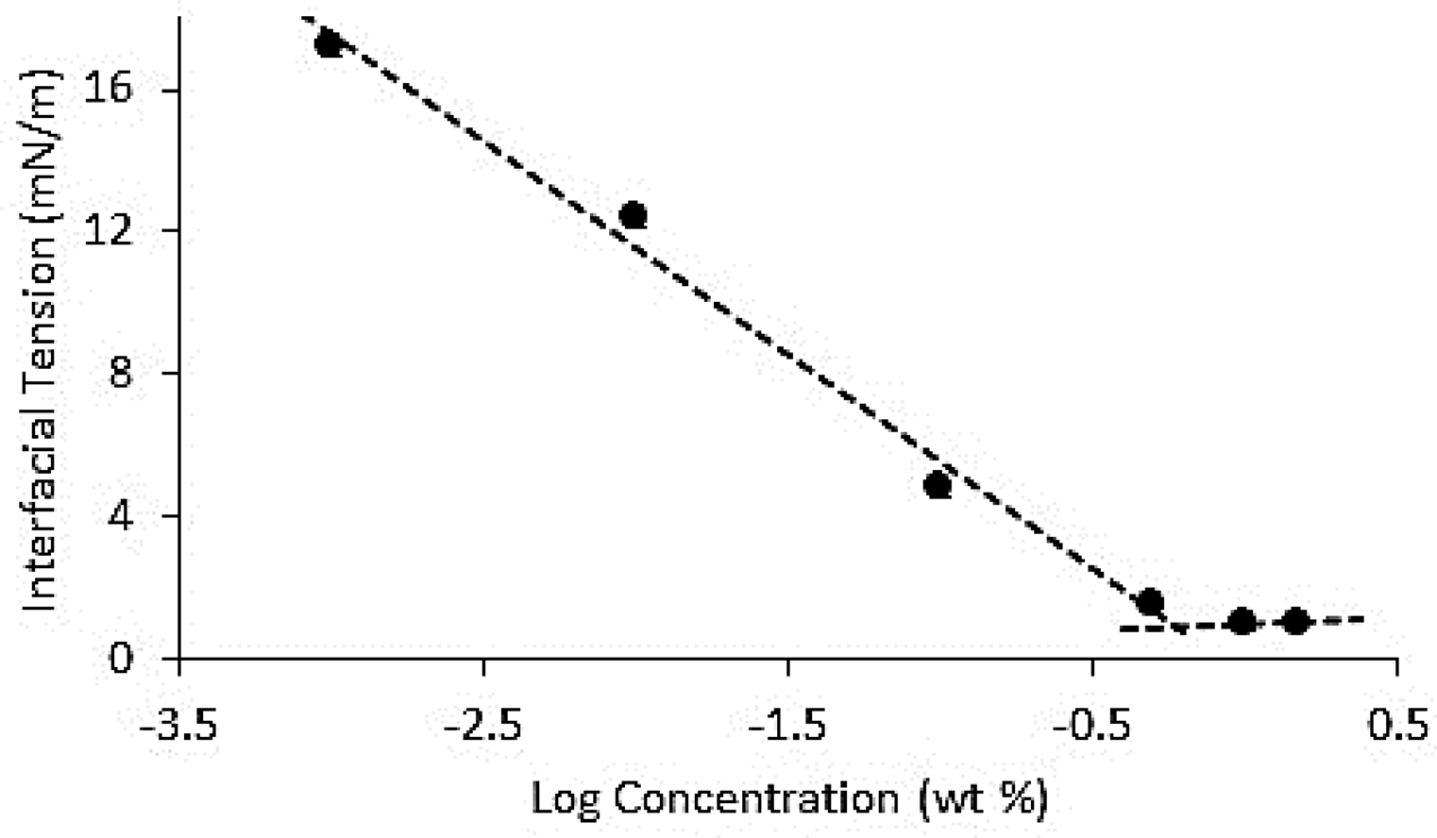
The Interfacial tension of canola oil/water system containing increasing concentrations of Span 80 in the oil phase The CMC of Span 80 is the point of intersection between the two slopes (~0.6%).

The CMC of Span 80 in a canola oil/water system was determined using the Wilhelmy plate method. The point where slope of the interfacial tension changed with increasing Span 80 concentration was due to interface saturation (Figure 6). The point of intersection of the two slopes was the CMC of the canola oil/water system (~0.6%). The CMC agrees well with data in Figure 4, which shows a dramatic increase in the release of NaCl from MSPE with between 0.5 and 0.7% Span 80 added to the emulsion. It is therefore suggested that the CMC of Span 80 added to the GMS-MSPE system was the threshold concentration above which increased destabilization phenomena were observed. Furthermore, destabilization of the MSPE appears to be one of the mechanisms to drive and modulate the release of the entrapped solute.

The native Pickering emulsions posed obvious problems due to their overly effective retention of the NaCl and Na citrate in the absence of a destabilizer. Previous efforts to displace interfacially-bound particles focused on dilution or pH variations met with limited success as particles in biphasic solutions remained attached to the interface even after phase separation [19–21]. Fujii *et al.* [22] reported on poly(4-vinyl pyridine)/silica particles that acted as pH-sensitive Pickering particles. Acidification of the aqueous phase resulted in protonation of the 4-vinyl pyridine residues, which increased the hydrophilicity of the particles and displaced them towards the aqueous phase. Via competitive adsorption between surfactants, Zhang *et al.* [23] reported emulsion destabilization by phase inversion when Span 80 was added to a W/O emulsion stabilized by synthetic clay nanoparticles.

For the present study, the addition of an oil phase amphiphile above the CMC appeared to actively promote the release of the active, providing an opportunity to modulate active solute release from controlled destabilization of the Pickering emulsions. This approach was likely related to significant changes in the three-phase contact angle and possibly the oil-water interfacial tension.

**Figure 7:**
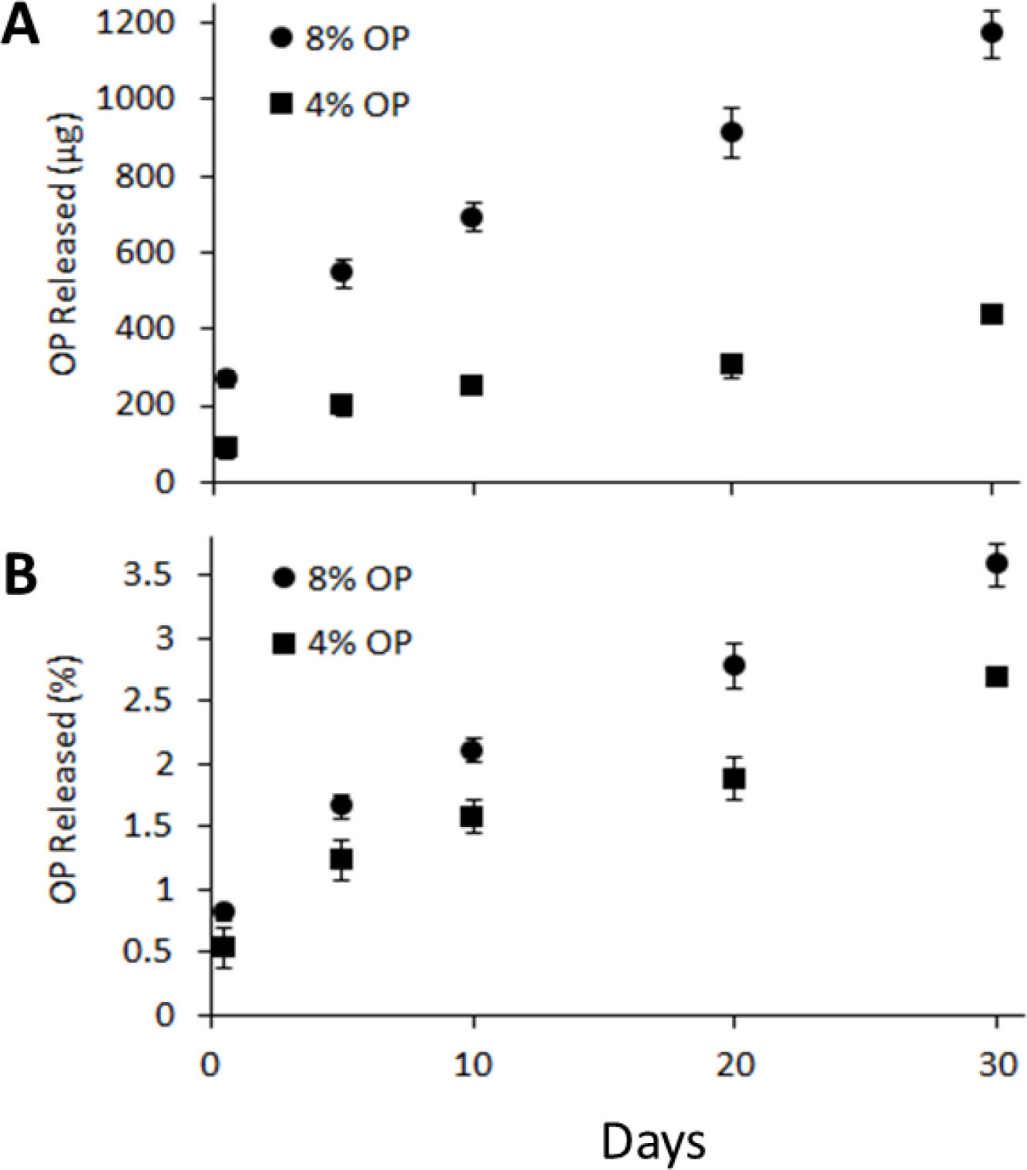
Release profile of OP from GMS-MSPE formulations at 37° C over 30 days, showing mass release (**A**) compared to percent release (**B**).

A potentially significant application for MSPE is in the sustained delivery of injectable therapeutics over extended-term. The demonstration of this potential was examined through the encapsulation, stability, retention and sustained release of OP. Previously, delivery of OP to a pancreatic tumor site over a 30-day period was demonstrated with poly(lactic-co-glycolic acid) (PLGA) cylindrical implants [8]. The goal in the present study was to replace a surgically implantable drug delivery vehicle with an injectable vehicle, providing similar dosing and release profiles at the tumor site, to that obtained with the PLGA implant. To that end, MSPE were loaded with varying concentrations of OP in the dispersed aqueous phase to examine release kinetics and assess the impact of drug loading on 30-day release profiles at 37°C for emulsions encapsulating OP at concentrations of 4 and 8% of the dispersed phase (Figure 7).

A slow, nearly linear sustained release of MSPE-encapsulated OP, was observed with less than 5% of the total drug loading being released over a 30 day period. This result was most similar to that obtained with sodium citrate which also showed less than 6% release over 30 days. More OP was released from emulsions containing 8% versus 4% OP (1170 μg vs. 430 μg respectively), which may be expected from emulsions containing double the amount of solute. Thus percent release was about the same between the two loading levels.

Release profiles from MSPE containing NaCl, Na citrate, and OP all showed stable, extended and nearly linear release profiles over 20–30 day periods, with minimal initial burst release. Thus, the total release of the active in these cases would take considerably longer than the 30 day experimental period. A preliminary experiment conducted to observe the stability of the MSPE beyond 30 days showed the continual release of OP for 70 days (data not shown).

Though drug delivery vehicles have been designed for sustained release applications over periods of several months, a significant objective of the present work was to develop a method to adjust release rate, such that most release would occur within a target of 30 days. It was demonstrated that Span 80 could promote emulsion instability, enhancing and potentially modulating NaCl release. The same approach was then tested with OP as the encapsulant.

**Figure 8:**
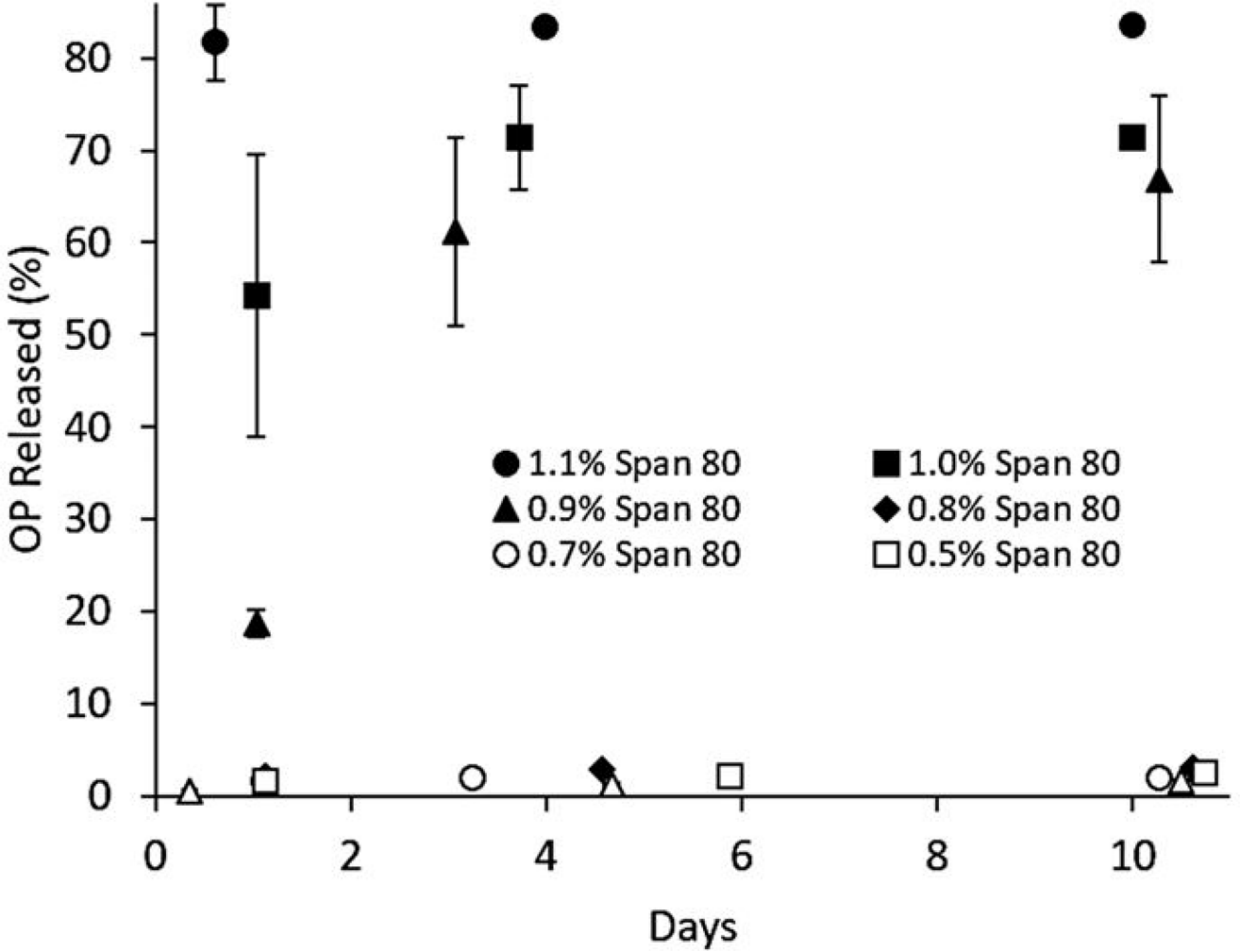
Sustained release of OP from MSPE formulations with varying amounts of Span 80 (wt% of oil phase) added post-emulsification Results are given as percent of total encapsulated OP.

The release profiles of OP from MSPE-stabilized emulsions in the presence of varying amounts of Span 80 are shown in Figure 8. Release profiles showed the minimal release of OP over ten days at Span 80 concentrations up to 0.8%. Beyond this critical concentration, sharp increases in release rates were observed, to an extent dependent on the Span 80 concentration. Between 4 and 5 days, release rates followed a rapid decay and plateauing as most OP had been released. At Span 80 concentrations below 0.8%, the release profile was monitored over 30 days, with no changes in the slopes of the release profiles observed. The entire OP released after 30 days was similar to the amounts shown in the absence of Span 80 in Figure 7.

The Span 80 threshold concentration enabling release of OP was 0.8%, similar to the CMC value determined in Figure 6 of 0.6% and similar to the critical Span 80 concentration of 0.7% needed to drive the release of NaCl from the same emulsion formulation.

Following 10 and 30 day release experiments, the remaining emulsions were gently heated to melt and remove remaining GMS, and to recover the residual OP. The total amount of OP released over the 10 or 30-day release experiment, combined with the mass of residual OP at the end of the experiment, totaled an average of 85% of the initial amount of OP encapsulated, which is a high recovery, demonstrating that non-released OP mostly remained within the emulsion. Previously, we have shown that up to 30% of the OP in a buffered solution will convert to oseltamivir carboxylate over 30 days through ester bond hydrolysis, and the extent of hydrolysis will depend on the particular buffer conditions [7]. That 85% of the OP is being accounted for at the end of an extended term release experiment further demonstrates the efficient encapsulation of the OP within these emulsions.

**Figure 9:**
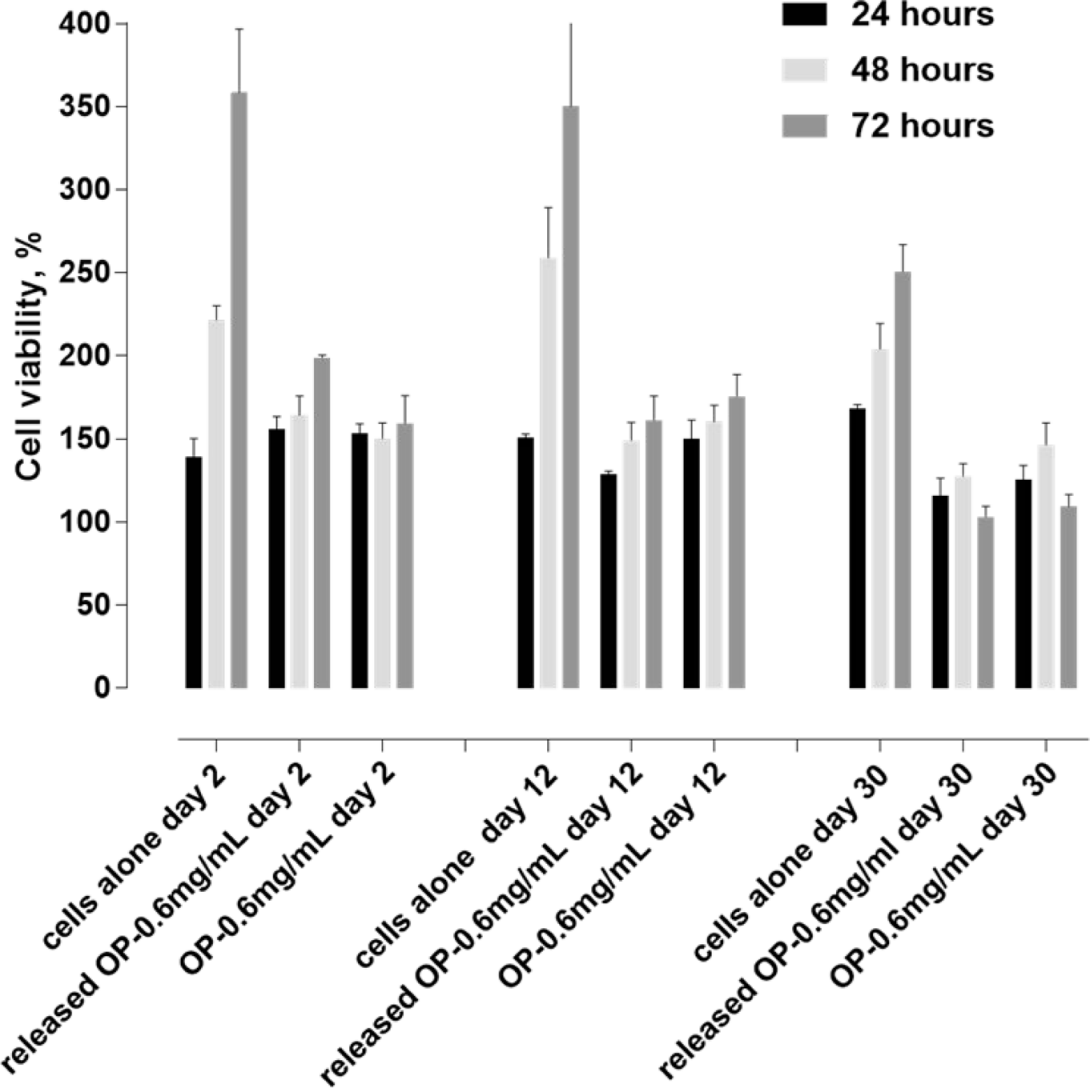
PANC-1 cell viability over 72 h, based on WST-1 assay following exposure of cells to OP released from MPSE at 2, 12 and 30 days Results are grouped into clusters of 3 bars for each time point of release. The first bar in each cluster represents cells exposed to added water plus media, to the same dilution extent as with samples containing OP supernatant. The second bar represents cells exposed to OP released from MSPE diluted to 0.6 mg/mL OP, and the third bar represents cells exposed to the fresh OP, also at 0.6 mg/mL. Cell viability in all cases is as a percent, in comparison to untreated, undiluted cells at time zero.

The efficacy of OP released from MSPE was tested against PANC-1 in culture using the WST-1 viability assay. The objective was to ensure that OP released over the experimental period continued to demonstrate activity against PANC-1. OP released from loaded MSPE (initially 12% OP in the aqueous phase and 1% Span 80 in the oil phase) was separated from an aqueous reservoir through a sterilizing 0.2 μm pore size membrane, enabling separation of the released OP from the emulsion into a small aqueous volume (2 mL). The OP supernatant was sampled, its concentration determined, and sample diluted in culture medium to achieve a consistent OP concentration of 0.6 mg/mL for all time points. Cell viability over 72 h exposure to OP sampled on release days 2, 12 and 30 is presented in Figure 9.

PANC-1 viability was significantly lower when exposed to released OP at all time points leading up to 30 days, in comparison to untreated cells, or cells cultured in diluted media (cells alone, first bar of each set). Also, there was no significant difference in the reduction in viability between PANC-1 exposed to OP released from the emulsion, and cells exposed to the same concentration of new OP. Thus, the OP being released for 30 days from the MSPE was as active regarding reducing cell viability, as was new OP.

A comparison can also be drawn to PANC-1 cultured in media diluted to the same extent as OP-based media but without OP present. At all time points, cell viability (growth) was observed over 72 h in the diluted media, and to a much more considerable extent than cells cultured in the presence of OP where any increase was either modest or not at all as was the case for day 30. Cell viability in diluted media showed some fluctuation over the 30 day timeframe, likely because the dilution was not the same from one-time point to the next. The media was diluted to the same extent as that required to reduce the OP concentration to 0.6 mg/mL for each sampling point. This approach ensured that cells were exposed to the same concentration of OP for samples collected on days 2, 12 and 30.

In parallel to that described above, an MSPE containing 1% Span 80 without OP was held for 30 days, and the supernatant sampled and tested for any effect of Span 80 on cell viability. There was no significant difference in cell viability (data not shown) between the 30 day sampled supernatant, and media diluted to the same extent, demonstrating that MSPE components including Span 80 did not affect cell viability.

The impact of direct exposure of PANC-1 to OP-loaded MSPE was examined in culture media, with MSPE containing 12% OP in the emulsified aqueous phase. The OP-MSPE was prepared, then vortex-mixed with culture media forming a secondary emulsion, which was then added to the culture well, containing cells and media. After 72 h, the emulsion mixture was removed from the well, and cells washed with fresh media before conducting a WST-1 cell viability assay.

**Figure 10:**
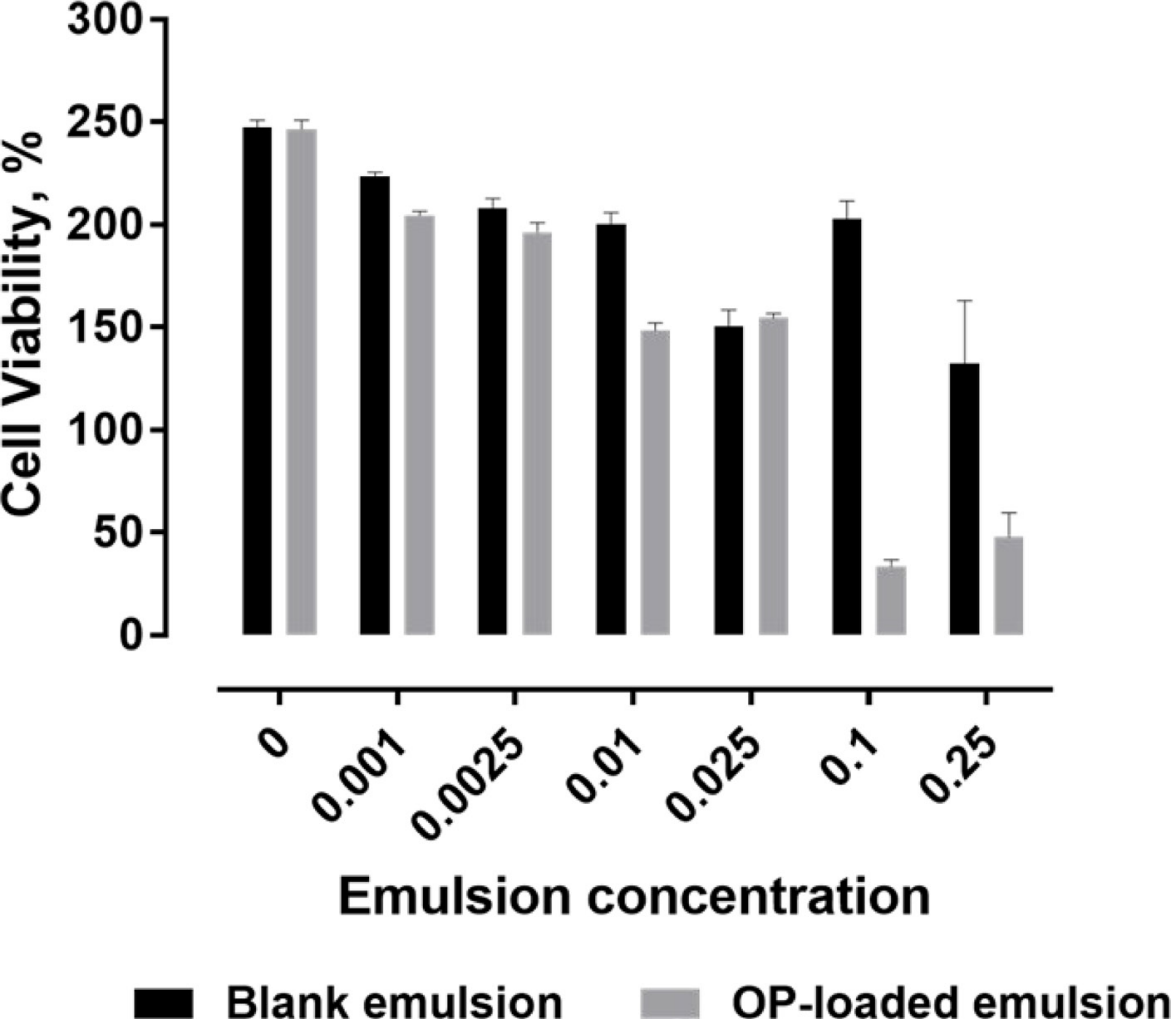
Cell viability determined by WST-1 assay, following 72 h exposure of PANC-1 to OP-loaded MSPE or blank-MSPE, as a percent in comparison to non-treated cells at time zero following 72 h exposure of PANC-1 to OP-loaded MSPE OP-MSPE are loaded with 12% OP in the aqueous phase. Emulsion concentration in the culture media is expressed as a mass fraction.

Data in Figure 10 compares cell viability after 72 h exposure to OP-loaded and blank-MSPE, expressed as percent of the viability of non-treated cells at time zero. For the case of 0 weight fraction of the emulsion, the cell viability had more than doubled over 72 h. Increasing the weight fraction of emulsion resulted in lower cell viability whether the MSPE contained OP or not. However, with one exception, cells exposed to OP-MSPE showed lower viability than cells exposed to empty emulsions, with the difference growing more substantial at the highest two emulsion concentrations. The most extensive reduction in cell viability compared to the blank-MSPE was observed at an emulsion weight fraction of 0.1. This difference would be due to the release of OP from the MSPE. An apparent reduction in cell viability, when exposed to blank emulsions, may merely be due to dilution effects. For the 0.1 weight fraction emulsion added to the cell media, the maximum concentration of OP in the media would be 2.6 mg/mL, which is well above the concentration used in the viability test presented in Figure 9, where concentrations of 0.6 mg/mL were shown to produce significant changes in cell viability. Concentrations as low as 0.3 mg/mL were also shown to have significant effects on PANC-1 cell viability in a previous study [16]. Although the amount of OP in the culture media that the cells were directly exposed to in the present case was not measured, it is clear that OP released from the MSPE is negatively impacting cell viability, and thus reaching therapeutic exposure levels over the 72 h period of release. Also, the reduced growth in the presence of blank-MSPE could be explained by a dilution effect, but in earlier presented results, it was shown that Span 80 did not affect cell viability. The effects observed with OP-MSPE on PANC-1 cell viability are due to the effective level of exposure to OP.

The potential for injectability of the viscous MSPE was demonstrated using syringe needles of varying diameter. A 18 G needle (838 μm internal diameter) showed good injectability, and a 20 G needle (603 μm internal diameter) showed fair injectability, both of which did not affect emulsion droplet size. Needles with higher gauge values showed poor injection characteristics.

## CONCLUSIONS

A proof of concept is provided for the use of MSPEs in the long-term release and delivery of active OP, as a therapeutic directed against human pancreatic cancer cells. The W/O formulation described in this report is simple, quick (1 h formulation), and uses equipment that is low cost and readily available. It is conducive to aseptic formulation and uses a small number of ingredients which are readily available and food/pharmaceutical acceptable. The MSPE can be formulated by personnel without specialized training, and the formulation methods involve no chemistry. The emulsions were shown to be injectable, and are made up of materials that are food grade and thus can be ultimately metabolized and eventually dispersed from the tumor injection site. The formulation shows promise for the long-term controlled delivery of a wide range of actives. In particular, these actives would include small molecular weight hydrophilic compounds that are typically difficult to encapsulate and retain in formulations with sustain release over extended periods of time. To this end, the therapeutic activity of OP against pancreatic cancer cells was demonstrated for a 30 day sustained release with virtually full encapsulation of the drug, and 30 day sustained activity, similar to that of the fresh drug. The challenge in the application of MSPE appears to be the inherent stability of the emulsion as the limiting factor for sustained release. Here, Span 80 was used as a destabilizer, modulating release, but the next phase of the research will be to look at a variety of the destabilization approaches to improve a tighter control over the duration of the release profile.

## MATERIALS AND METHODS

### Materials

Canola oil was purchased from a local retailer. Typical composition and physical properties can be obtained from Canola Council of Canada [24, 25]. In a previous study, the acid value of this canola oil was determined to be 0.2 mg KOH/g oil [14].

Distilled monoglycerides containing ≥ 90% glycerol monostearate (GMS, CAS # 123-94-4) were obtained from Corbion (Lenexa, KS, USA). This product was used as the primary stabilizer in all Pickering emulsions prepared. Sorbitan monooleate (Span^®^ 80, CAS # 1338-43-8, HLB 4.3) was obtained from Sigma Aldrich. Span^®^ 80 is a sorbitan ester-containing one fatty acid chain. The fatty acid composition was ≥ 60% oleic acid, with the balance being primarily linoleic, linolenic, palmitic, and palmitoleic acids. Sodium chloride (CAS # 7647-14-5, MW 58.44 g/mol, ≥ 99%) and sodium citrate dihydrate (CAS # 6132-04-3, dry MW 258.06 g/mol, ≥ 99%) were obtained from Sigma Aldrich. Oseltamivir phosphate (CAS # 204255-11-8, MW 410.4 g/mol, ≥ 99%) was obtained from Hangzhou DayangChem Co. Ltd (Hangzhou City, People's Republic of China).

Spectra/Por^®^ 3 regenerated cellulose dialysis membranes with a molecular weight cut-off (MWCO) of 3.5 kD and 11.5 mm diameter were obtained from Spectrum Labs (Rancho Dominguez, CA, USA). Membranes were cut to the desired length and hydrated in distilled water for 30 minutes before use.

Distilled water with an average conductivity of 1.4 μS/cm was used for all experiments. HPLC grade methanol (CAS # 67-56-1, ≥ 99.9%) was obtained from Fisher Scientific, and 0.1 ± 0.07 v% trifluoroacetic acid (TFA, CAS# 76-05-1) in water solution was obtained from Sigma Aldrich.

Human pancreatic cancer cells (PANC1, ATCC^®^ CRL-1469™) were obtained from the American Type Culture Collection (Manassas, VA, USA). Cells were grown in a 37°C incubator with 5% CO_2_ and cultured in Dulbecco's Modified Eagle Medium containing 10% fetal bovine serum and 5 μg/mL Plasmocin™ [16].

### Formation of Pickering emulsion

GMS-stabilized Pickering emulsions (GMS-MSPE or MSPE) were prepared using a formulation method as illustrated in Figure 1. An oil phase was prepared by mixing the Pickering stabilizer (GMS) with canola oil to give a concentration of 4 wt% GMS in oil. An aqueous phase was prepared by mixing the solute to be encapsulated (sodium chloride, sodium citrate or OP) with distilled water at a concentration of 4 wt% solute in water. Both mixtures were warmed separately to 75°C to melt the GMS. A coarse emulsion was then produced by adding the aqueous (dispersed) phase to the oil (continuous) phase to yield an emulsion containing 80 wt% oil phase and 20 wt% aqueous phase. The stirrer speed was increased to 1000 rpm to produce a W/O emulsion, and the temperature was maintained at 75°C.

A fine emulsion was produced by processing the coarse emulsion with a high-shear mixer (Janke & Kunkel Ultra-Turrax, IKA^®^ Works, Inc., Wilmington, NC, USA) operating at 20000 rpm, with an appropriately sized dispersing tool selected based on the volume of the emulsion. The coarse emulsions were mixed for at least two minutes, or until a homogeneous appearance was obtained. The temperature was controlled by immersing the emulsification vessel in water at 75°C. Following emulsification, the vessel containing the fine emulsion was quench-cooled in a room temperature water bath. Stirring was provided by a magnetic stirrer operating at 600 rpm. The stirrer speed was increased as the emulsion cooled and became more viscous, to a maximum of 850 rpm. Emulsions were mixed during cooling for at least 2 h to ensure complete solidification of the stabilizer on the emulsion droplet surface. Samples were stored at room temperature for several hours to ensure stability before being used in any experiment.

### Solute release profiles

Various approaches for determining release kinetics from W/O emulsions were evaluated, any one of which can impact on the observed release kinetics. It was our objective to simulate *in vivo* conditions. Also, there needed to be an external aqueous phase as a sink, into which the water-soluble solute could be released, as would be the case in the presence of physiological fluids. A dialysis membrane method was devised and selected as the most suitable method to measure the sustained release of solute actives from the MSPE. A dialysis membrane with MWCO of 3.5 kD was filled partially with distilled water as the sink (1 mL), and partially with MSPE (1 mL). It was observed that the viscous MSPE did not disperse into the aqueous sink phase within the membrane, and the sink phase did not appear to destabilize the emulsion, even in the presence of gentle mixing. The membrane was sealed and immersed in distilled water to a total water volume of 30 mL. A shaker plate operating at low speed provided mixing. The membrane MWCO was selected to ensure that the solutes of interest were able to diffuse through readily, but that the emulsion was retained. Preliminary tests indicated that membranes with an MWCO of 3.5 kD or higher did not pose a significant diffusional barrier for the encapsulated solutes.

At the beginning of the release experiments, vessels containing membrane-enclosed emulsions were sealed with aluminum foil and mixed gently at room temperature or 37°C. The water phase external to the dialysis membrane was sampled at regular intervals to determine the concentration of solutes of interest. Any volume removed was replaced with water. NaCl and sodium citrate releases were quantified by solution conductivity using an Oakton Cono 6+ conductivity meter (Oakton Instruments, Vernon Hills, IL, USA) with automatic temperature compensation. Background conductivity was subtracted from all measurements during the release experiment. Standard solutions and calibration plots were prepared for the solutes for each release experiment, to determine the mass of solute released from the conductivity measurements.

### Oseltamivir phosphate (OP) analysis

OP release from the MSPE was quantified using a reverse phase HPLC. A 1260 Infinity HPLC unit with poroshell 120 SB-C18 threaded column (Agilent Technologies, Santa Barbara, CA, USA) of dimension 4.6 × 50 mm with 2.7 μm packing material was used to identify OP by its residence time in the column (2.7–3.0 min). The mobile phase was composed of 60 v% methanol and 40 v% 0.04 mol/L acetate buffer solution at pH 5.2. The acetate buffer was prepared by dissolving the required amount of ammonium acetate in a 0.1 v% TFA in water solution. Corrections to the pH of the resulting solution were made by adding small amounts of 0.1 M sodium hydroxide. The mobile phase flow rate was set to 1 mL/min, and the column temperature was maintained at 25°C. The retention time of OP in the column was approximately 3 min, and UV absorbance of the eluting OP was measured at 230 nm and compared to calibration standards based on known quantities of pure OP for quantification.

### Post-release solute recovery

After release experiments were concluded, residual emulsion samples were recovered in a flask, and excess water caught up with the emulsion removed by evaporation for 24 h with gently circulating air. Distilled water (20 mL) was added to the emulsion, which was then warmed with mixing to 75°C to melt the GMS Pickering stabilizer and release any remaining encapsulated solute. The emulsion was then cooled to room temperature, and a sample of the aqueous subnatant withdrawn by syringe was passed through a 0.2 μm syringe filter to remove colloidal material. This filtrate was tested by the appropriate method to determine the total mass of solute remaining in the extracted emulsion samples, and periodically on samples stored over extended periods of time to assess stability.

### Emulsion characterization

ImageJ software (National Institute of Health) was used to analyze images obtained from photomicrographs of emulsions taken with a Leitz Dialux 20 microscope (Ernst Leitz Wetzlar GmbH, Germany). The software was calibrated using microscope software-generated scale bars or measurements taken from images of an etched hemocytometer grid at an appropriate magnification. Droplet size distributions are reported based on a minimum of 50 droplets measured per sample. In the case of irregularly shaped droplets, the diameter was recorded as the length of a horizontal line passing through the center of mass in standard orientation.

### Critical Micelle Concentration (CMC) determination

The interfacial tension between canola oil and water was measured using a TensioCAD-M^®^ (CAD Instruments, Les Essarts-le-Roi, France) tensiometer with the Wilhelmy plate method using a platinum-iridium plate. The plate was immersed in the water phase, and the oil phase carefully layered on top. After 30 min equilibration, the weighing sensor was zeroed, and the plate slowly pulled through the liquid-liquid interface until detachment. The maximum force required to draw the plate through the interface yielded the interfacial tension, from which the critical micelle concentration (CMC) of an added surfactant (Span 80) was calculated.

### Cell viability assay

The effect of MSPE release of OP on PANC-1 viability was assessed using the WST-1 assay [16], which measures the absorbance at 420 nm of the reduced derivative of a tetrazolium salt through the action of mitochondrial enzymes. The degree of conversion (intensity of color) is directly correlated with the amount of metabolic activity in a cell culture.

**Figure 11:**
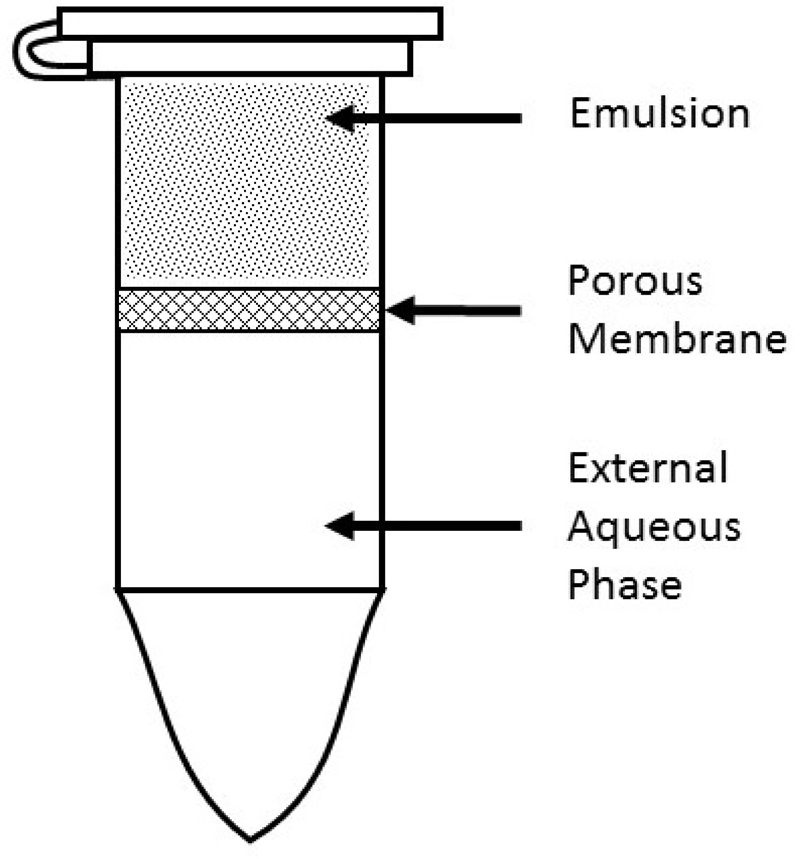
Release apparatus used to capture OP released from MSPE for use in cell viability assays

Separation of MSPE-releasing OP from the aqueous-receiving reservoir was possible with a 2 mL centrifuge tube partitioned with a 0.2 μm porous filter. The lower portion of the tube was the receiving reservoir filled with 1 mL water, and the upper portion above the membrane contained 450 μL of MSPE-releasing OP ultimately through the membrane, and into the receiving reservoir as illustrated in Figure 11. The upper compartment was removable enabling sampling of the lower layer (subnatant), with sterility maintained by the filter separating the two compartments. Sampled subnatant was replaced with distilled water at each sampling time point over the 30-day release period. The release of OP took place at 37°C unless stated otherwise. A portion of this subnatant was analyzed for OP, and the remainder mixed with cell culture media such that the OP concentration was diluted to a standard value (0.6 mg/mL) for cell viability testing. Working solutions were normalized by diluting the untreated control media to the same extent as that of the sample wells.

PANC-1 cells were added to a welled microplate at a density of 5000 cells/well and incubated overnight in media mixed with the OP subnatant. The control wells were untreated cells at time zero. The WST-1 reagent was added to each well 2 h before measuring absorbance at 420 nm with a Spectramax M2 microplate reader (Molecular Devices Corporation, Sunnyvale, CA, USA). Measurements were taken at 24, 48, and 72 h, and compared to the control wells to determine relative cell viability under treatment.

The effect of direct contact between PANC-1 cells and MSPE was accessed by mixing blank and OP-MSPE with cell culture media using a vortex mixer at dilutions between 0.1 – 25 wt% emulsion in media. This protocol formed a secondary emulsion where oil droplets containing Pickering stabilized water droplets were dispersed in cell culture media. Aliquots of these mixtures were added to a welled microplate that had been seeded with PANC-1 cells at a density of 5000 cells/well and incubated for 72 h. After treatment, the MSPE was removed and each well washed with fresh cell culture media. A WST-1 assay was then carried out as previously detailed to compare the cell viability of treated wells to that of control. Blank measurements were conducted using emulsions with no drug added, in addition to untreated wells for comparison. Turbidity was addressed by washing the emulsion off the plated cells before assay or microscopy. All wells received the same treatment otherwise.

### Data analysis

Unless otherwise stated, experiments were carried out in triplicate, and experimental values reported as the sample mean ± standard error of the mean. Plotted error bars represent standard error of the mean. Statistical analysis was carried out using Microsoft Excel spreadsheet software. Unpaired *t*-test, *F*-test, and one-way ANOVA were used to analyze for significant differences between sample means and variances. Strong evidence against the null hypothesis was considered for *p* < 0.05. Throughout, all concentrations given as percent are in wt%, unless otherwise noted.

## References

[1] Pickering SU (1907). CXCVI.-Emulsions. J Chem Soc Trans.

[2] Simovic S, Prestidge CA (2007). Nanoparticle layers controlling drug release from emulsions. Eur J Pharm Biopharm.

[3] Liu F, Tang CH (2013). Soy protein nanoparticle aggregates as pickering stabilizers for oil-in-water emulsions. J Agric Food Chem.

[4] Frelichowska J, Bolzinger MA, Valour JP, Mouaziz H, Pelletier J, Chevalier Y (2009). Pickering w/o emulsions: drug release and topical delivery. Int J Pharm.

[5] Frelichowska J, Bolzinger MA, Pelletier J, Valour JP, Chevalier Y (2009). Topical delivery of lipophilic drugs from o/w Pickering emulsions. Int J Pharm.

[6] Simovic S, Ghouchi-Eskandar N, Prestidge CA (2011). Pickering emulsions for dermal delivery. J Drug Deliv Sci Technol.

[7] Allison Logan S, Brissenden AJ, Szewczuk MR, Neufeld RJ (2017). Combinatorial and sequential delivery of gemcitabine and oseltamivir phosphate from implantable poly(d,l-lactic-co-glycolic acid) cylinders disables human pancreatic cancer cell survival. Drug Des Devel Ther.

[8] Hrynyk M, Ellis JP, Haxho F, Allison S, Steele JA, Abdulkhalek S, Neufeld RJ, Szewczuk MR (2015). Therapeutic designed poly (lactic-co-glycolic acid) cylindrical oseltamivir phosphate-loaded implants impede tumor neovascularization, growth and metastasis in mouse model of human pancreatic carcinoma. Drug Des Devel Ther.

[9] Haxho F, Neufeld RJ, Szewczuk MR (2016). Neuraminidase-1: a novel therapeutic target in multistage tumorigenesis. Oncotarget.

[10] Gilmour AM, Abdulkhalek S, Cheng TS, Alghamdi F, Jayanth P, O’Shea LK, Geen O, Arvizu LA, Szewczuk MR (2013). A novel epidermal growth factor receptor-signaling platform and its targeted translation in pancreatic cancer. Cell Signal.

[11] Haxho F, Allison S, Alghamdi F, Brodhagen L, Kuta VE, Abdulkhalek S, Neufeld RJ, Szewczuk MR (2014). Oseltamivir phosphate monotherapy ablates tumor neovascularization, growth, and metastasis in mouse model of human triple-negative breast adenocarcinoma. Breast Cancer (Dove Med Press).

[12] Abdulkhalek S, Geen OD, Brodhagen L, Haxho F, Alghamdi F, Allison S, Simmons DJ, O’Shea LK, Neufeld RJ, Szewczuk MR (2014). Transcriptional factor snail controls tumor neovascularization, growth and metastasis in mouse model of human ovarian carcinoma. Clin Transl Med.

[13] Kapishon V, Allison S, Whitney RA, Cunningham MF, Szewczuk MR, Neufeld RJ (2016). Oseltamivir-conjugated polymeric micelles prepared by RAFT living radical polymerization as a new active tumor targeting drug delivery platform. Biomater Sci.

[14] Nadin M, Rousseau D, Ghosh S (2014). Fat crystal-stabilized water-in-oil emulsions as controlled release systems. Lebenson Wiss Technol.

[15] Haxho F, Haq S, Szewczuk MR (2018). Biased G protein-coupled receptor agonism mediates Neu1 sialidase and matrix metalloproteinase-9 crosstalk to induce transactivation of insulin receptor signaling. Cell Signal.

[16] O’Shea LK, Abdulkhalek S, Allison S, Neufeld RJ, Szewczuk MR (2014). Therapeutic targeting of Neu1 sialidase with oseltamivir phosphate (Tamiflu®) disables cancer cell survival in human pancreatic cancer with acquired chemoresistance. Onco Targets Ther.

[17] Avranas A, Malasidou E, Mandrazidou I (1998). Adsorption of cetyldimethylbenzylammonium chloride on octane emulsions droplets: the effect of the presence of Tween 80. J Colloid Interface Sci.

[18] Vílchez A, Rodríguez-Abreu C, Menner A, Bismarck A, Esquena J (2014). Antagonistic effects between magnetite nanoparticles and a hydrophobic surfactant in highly concentrated Pickering emulsions. Langmuir.

[19] Yan Y, Masliyah JH (1993). Solids-stabilized oil-in-water emulsions: scavenging of emulsion droplets by fresh oil addition. Colloids Surf A Physicochem Eng Asp.

[20] Zeng X, Osseo-Asare K (2004). Partitioning behavior of silica in the Triton X-100/dextran/water aqueous biphasic system. J Colloid Interface Sci.

[21] Xu H, Melle S, Golemanov K, Fuller G (2005). Shape and buckling transitions in solid-stabilized drops. Langmuir.

[22] Fujii S, Read ES, Binks BP, Armes SP (2005). Stimulus-responsive emulsifiers based on nanocomposite microgel particles. Adv Mater.

[23] Zhang J, Li L, Wang J, Sun H, Xu J, Sun D (2012). Double inversion of emulsions induced by salt concentration. Langmuir.

[24] Przybylski R (2001). Canola oil: physical and chemical properties. Canola oil technical information kit.

[25] Malcolmson L, Vaisey-Genser M (2001). Canola oil: performance properties of canola oil. Canola oil technical information kit.

